# A 10-Year Retrospective Cohort Study of Endometrial Cancer Outcomes and Associations with Lymphovascular Invasion: A Single-Center Study from Germany

**DOI:** 10.3390/diagnostics14151686

**Published:** 2024-08-04

**Authors:** Alexandra Nienhaus, Rahavie Rajakulendran, Elena Bernad

**Affiliations:** 1 Doctoral School, “Victor Babes” University of Medicine and Pharmacy, Eftimie Murgu Square 2, 300041 Timisoara, Romania; alexandra.nienhaus@umft.ro; 2 Department of Obstetrics and Gynaecology “Augusta Krankenanstalt” Bochum, Bergstr. 26, 44807 Bochum, Germany; 3 Orthopaedics and Trauma Surgery, St. Josef-Hospital and Catholic Hospital Bochum, Gudrunstraße 56, 44791 Bochum, Germany; 4 Department of Obstetrics and Gynecology, “Victor Babes” University of Medicine and Pharmacy, Eftimie Murgu Square 2, 300041 Timisoara, Romania; bernad.elena@umft.ro; 5 Center for Laparoscopy, Laparoscopic Surgery and In Vitro Fertilization, “Victor Babes” University of Medicine and Pharmacy, Eftimie Murgu Square 2, 300041 Timisoara, Romania

**Keywords:** oncology, gynecology, endometrial cancer

## Abstract

This 10-year retrospective cohort study at a single-center clinic in Germany aimed to analyze the outcomes of endometrial cancer patients and explore the impact of lymphovascular invasion (LV) on patient outcomes and disease-free survival (DFS). Identifying correlations among demographic data, tumor characteristics, treatment modalities, and survival outcomes could enhance patient management and improve survival rates. The study encompassed patients diagnosed and treated for endometrial cancer from January 2010 to December 2020. Clinical and pathological data were extracted from medical records for 311 patients, focusing on variables such as age, histological type, tumor grade, type of surgical treatment, and adjuvant therapies. Survival analysis was conducted using the Kaplan–Meier method and multivariate Cox proportional hazard models to identify factors independently associated with survival. The study demonstrated that lymphovascular invasion significantly impacted survival outcomes on Kaplan–Meier analysis (log-rank *p*-value = 0.0058). Patients with LV showed a marked decrease in DFS compared to those without LV invasion, with a median DFS of 3.2 years and a hazard ratio of 2.18 (95% CI: 1.56–3.04, *p* < 0.001). Furthermore, high-grade tumors and p53 positivity were strongly associated with reduced DFS, with hazard ratios of 1.93 (*p* = 0.001) and 2.11 (*p* < 0.001), respectively. Patients with distant metastasis exhibited the most significant decline in survival, with a hazard ratio of 5.56 (95% CI: 2.45–10.18, *p* < 0.001). Despite comprehensive surgical and adjuvant therapies, these high-risk factors dictated poorer outcomes. The presence of lymphovascular invasion, high-grade tumors, and genetic markers like MSI and p53 are pivotal in predicting the course of endometrial cancer. This study underscores the necessity for aggressive management strategies in patients exhibiting these high-risk features to potentially improve prognosis and survival outcomes. The findings advocate for enhanced therapeutic strategies tailored to the biological behavior of the tumor, thereby aiming to elevate the overall survival rates for women diagnosed with endometrial cancer.

## 1. Introduction

Endometrial cancer remains the most prevalent gynecological malignancy in developed nations, with an increasing incidence attributed to rising obesity rates and an aging population [1,2]. In Germany, endometrial cancer accounts for approximately 5% of all new cancer cases among women, with an estimated 11,000 diagnoses annually based on recent data [3]. This malignancy of the uterus is primarily managed through surgical intervention and supplemented by adjuvant therapies such as radiotherapy and chemotherapy, depending on the histological subtype and stage at diagnosis [4,5].

The classification and prognosis of endometrial cancer are significantly influenced by the histopathological evaluation, which distinguishes between type I (endometrioid) tumors, often linked to excess estrogen and favorable prognosis, and type II (non-endometrioid) tumors, which are less common, but more aggressive [6,7,8]. Recent advancements in molecular profiling have further refined prognosis and treatment strategies, highlighting the heterogeneity of this disease. For example, the presence of mismatch repair (MMR) defects, microsatellite instability (MSI), and mutations in genes like PTEN, PIK3CA, and p53 have been correlated with distinct patterns of disease progression and response to treatment [9,10].

Despite these advancements, long-term outcomes for endometrial cancer patients have stagnated, particularly among those diagnosed with advanced-stage or higher-grade tumors [11,12,13]. Recent European studies have shown variable 5-year survival rates, from over 90% for localized disease to less than 20% for cancers with distant metastases [14,15,16]. Such disparities underscore the importance of ongoing research to understand the factors contributing to these differences and to develop more effective, personalized treatment protocols.

The primary objective of this retrospective study was to analyze the outcomes of endometrial cancer patients treated over the last decade at a single-center clinic in Germany. By examining a range of factors, including demographic data, tumor characteristics, treatment modalities, and survival outcomes, this study aimed to identify patterns and trends that could improve patient management. The hypothesis guiding this investigation is that initial presentation with lymphovascular invasion is associated with worsened patient outcomes and a lower disease-free survival (DFS). This analysis will contribute to the body of knowledge necessary to enhance therapeutic strategies and ultimately improve survival rates for women diagnosed with this condition.

## 2. Materials and Methods

### 2.1. Study Design and Ethics

This retrospective cohort study investigated endometrial cancer outcomes over a ten-year period at a single-center clinic in Germany. The study included patients diagnosed and treated for endometrial cancer between January 2010 and December 2020. Data were extracted from medical records, and included demographic information, clinical and pathological characteristics, treatment details, and follow-up outcomes. The study was conducted following approval from the institutional review board (IRB) of the clinic, ensuring adherence to ethical standards as per the Declaration of Helsinki and the EU Good Clinical Practice Directive (2005/28/EC). Prior to data analysis, all patient identifiers were removed to maintain confidentiality. As this is a retrospective analysis of existing data, the requirement for informed consent was waived by the IRB.

### 2.2. Patient Selection and Definitions

Patients included in the study were those with a confirmed histopathological diagnosis of endometrial cancer. Exclusion criteria were incomplete medical records, patients who were lost to follow-up immediately after initial diagnosis, and those who declined treatment. The final cohort was stratified based on histological subtypes, stage at diagnosis, and treatments received, facilitating comparative analyses across different demographic and clinical subgroups.

In this study, “recurrence” refers to the reappearance of endometrial cancer, either at the original site or as distant metastasis after a period of documented complete remission. It is confirmed by any of the following criteria: positive findings on clinical examination, imaging studies indicating new tumor growth, or histopathological evidence of malignant cells. The date of recurrence is recorded as the first date on which any of these criteria is met following the end of primary treatment. This definition is employed to determine disease-free survival, which is calculated from the end of primary treatment until the date of recurrence or last known follow-up if no recurrence occurs.

In our study, the distinction between lymphovascular invasion and venous invasion was rigorously established through histopathological evaluation. LV was identified when cancer cells were observed within both lymphatic channels and venous vessels, while venous invasion was specifically noted when cancer cells were exclusively found within venous structures, irrespective of lymphatic involvement. This differentiation was facilitated by employing immunohistochemical staining techniques, such as D2-40 for lymphatic endothelium and CD31 or CD34 for blood vessel endothelium, which enhance the visualization of the respective vascular structures under microscopic examination.

### 2.3. Data Collection

Data collection involved a comprehensive review of electronic health records and patient charts to extract relevant information. Variables collected included age at diagnosis, body mass index (BMI), histological type, grade of the tumor, stage at presentation, type of surgical treatment, adjuvant therapies (radiotherapy and chemotherapy), recurrence, and survival status at last follow-up.

### 2.4. Statistical Analysis

Statistical analyses were performed using SPSS software (version 25.0). Descriptive statistics such as means, medians, and standard deviations were used to summarize continuous variables, while frequencies and percentages described categorical variables. Survival analyses were conducted using the Kaplan–Meier method, and differences in survival rates across different subgroups were assessed using the log-rank test. Multivariate Cox proportional hazard models were used to identify factors independently associated with survival, adjusting for potential confounders. A *p*-value of less than 0.05 was considered statistically significant.

### 2.5. Follow-Up

Follow-up data were collected from the date of initial diagnosis to the date of last contact or death. The median follow-up time was calculated using the reverse Kaplan–Meier method. The primary endpoint of the study was overall survival, defined as the time from diagnosis to death from any cause. Secondary endpoints included disease-free survival, measured from the date of end of primary treatment to the date of recurrence or last follow-up.

## 3. Results

In this retrospective cohort study examining endometrial cancer outcomes and associations with lymphovascular invasion (LV), the analysis of background characteristics of 311 patients (261 without LV invasion and 50 with LV invasion) showed no significant differences in age or comorbid conditions between the two groups. The mean age of patients without LV invasion was 67.0 years (SD = 11.1) and 68.2 years (SD = 13.1) for those with LV invasion, yielding a *p*-value of 0.726. Additionally, when categorized by age, the distribution across different age groups (18–40 years, 40–60 years, and >60 years) did not significantly differ between the groups either (*p*-value = 0.185), indicating that age was not a determining factor in the presence of LV invasion.

Furthermore, the prevalence of comorbidities such as cardiovascular disease, diabetes, and obesity was assessed among the patients. There were no statistically significant differences in the incidence of these comorbidities between patients with and without LV invasion, with *p*-values uniformly above 0.7. Specifically, cardiovascular disease was present in 8.1% of patients without LV invasion versus 2.0% with LV invasion, diabetes appeared in 6.5% versus 4.0%, and obesity was reported in 10.3% versus 6.0%, respectively (Table 1).

The presence of microsatellite instability (MSI) and p53 positivity showed marked differences, with MSI present in 18.0% of patients with LV invasion and 8.4% of those without (*p* = 0.038). Similarly, p53 positivity was significantly higher in patients with LV invasion (14.0%) versus those without (3.4%), yielding a *p*-value of 0.002. These genetic markers indicate a potential link between molecular characteristics and the presence of LV invasion in endometrial cancer.

Surgical management also differed significantly between the groups, particularly in the choice of surgical access and outcomes regarding residual tumor after surgery. Patients with LV invasion were more likely to undergo total abdominal hysterectomy (36.00% vs. 25.29%) and less likely to receive laparoscopic hysterectomy (44.00% vs. 64.37%), with a significant *p*-value of 0.001. In terms of surgical access, a substantial portion of LV invasion patients required abdominal access (44.00% vs. 17.24%, *p* < 0.001), correlating with higher rates of residual tumors post-surgery. Notably, the proportion of patients with no residual tumor (R0) was significantly lower in the LV invasion group (62.00% vs. 89.27%), and the presence of microscopic (R1) and macroscopic (R2) residual tumors was considerably higher in this group (R1: 18.00%, R2: 14.00% vs. R1: 1.15%, R2: 0.77%), all with *p*-values below 0.001 (Table 2).

The staging and grading of endometrial cancer in the 311 patients displayed significant differences based on the presence of lymphovascular invasion, with dramatic contrasts in pathological node status, tumor grade, venous invasion, and distant metastasis. Grading of tumors revealed that 64.00% of patients with LV invasion had high-grade tumors (grades 3–4), in contrast to only 25.77% in the non-invasion group, demonstrating a significant correlation between LV invasion and higher tumor grade (*p* < 0.001). The number of lymph nodes removed during surgery showed no significant difference between the groups (mean ± SD: 9.39 ± 16.55 for no invasion vs. 14.43 ± 18.35 for LV invasion, *p* = 0.320), suggesting that the extent of surgical intervention in terms of lymph node removal was not influenced by the presence of LV invasion, as seen in Table 3.

Notably, a lower percentage of patients with LV invasion underwent a second surgery (66.67%) compared to those without LV invasion (78.95%), with a statistically significant *p*-value of 0.047. Furthermore, the occurrence of intraoperative and immediate postoperative complications was significantly higher in the LV invasion group (60.00%) versus the non-invasion group (39.08%), with a *p*-value of 0.014. This pattern extended to adjuvant treatments, where a significant difference in the administration of radiotherapy and chemotherapy was observed. Patients with LV invasion were more likely to receive both radiotherapy (60.00% Yes vs. 39.08% Yes) and chemotherapy (57.14% Yes vs. 22.94% Yes), with *p*-values of 0.003 and less than 0.001, respectively, underscoring the more aggressive treatment approach necessitated by the higher-risk profile associated with LV invasion.

**Figure 1:**
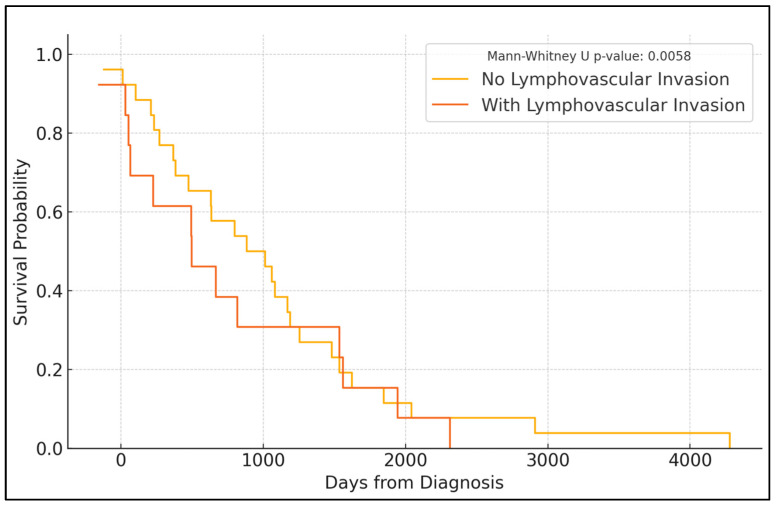
Kaplan–Meyer analysis for PFS among patients with endometrial cancer with and without lymphovascular invasion.

Further disparities were found in the follow-up progression-free survival (PFS) rates and overall patient outcomes over a five-year period. The PFS rates consistently showed that patients with LV invasion had a lower probability of remaining disease-free over the years, with a significant decline from 84.6% in year 1 to 50.8% by year 5, compared to those without LV invasion, who started at 95.1% and decreased to 74.3% (*p* = 0.001). Moreover, the mortality rate was significantly higher in the LV invasion group (40.48%) compared to the non-invasion group (17.59%), with a *p*-value of less than 0.001. The recurrence rates, however, did not show a statistically significant difference (17.95% in the LV group vs. 14.78% in the non-LV invasion group, *p* = 0.738), suggesting that while LV invasion is associated with higher initial aggressiveness and poorer initial outcomes, it does not necessarily predict a higher rate of cancer recurrence (Table 4). The Kaplan–Meier analysis (Figure 1) showed a significantly higher PFS among patients without initial LV invasion (log-rank *p*-value = 0.0058).

Lymphovascular invasion emerged as a particularly detrimental factor, with affected patients having a median DFS of only 3.2 years and a hazard ratio of 2.18, indicating more than double the risk of disease recurrence or progression compared to patients without this feature (95% CI: 1.56–3.04, *p*-value < 0.001). Other negative prognostic factors included the presence of high-grade tumors (grade 3–4), which correlated with a shorter median DFS of 2.8 years and a hazard ratio of 1.93 (95% CI: 1.42–2.61, *p*-value = 0.001), and microsatellite instability (MSI), which further compounded the risk with a hazard ratio of 2.67 (95% CI: 1.18–7.36, *p*-value = 0.004).

**Figure 2:**
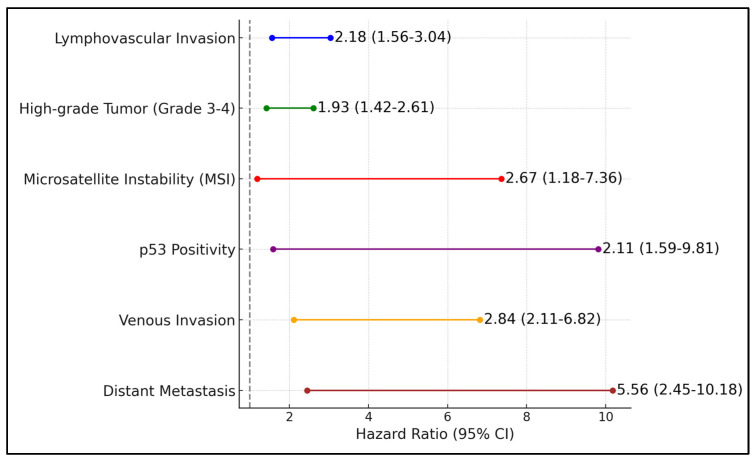
Negative factors for PFS in patients with endometrial cancer.

Additional genetic markers and tumor characteristics such as p53 positivity and venous invasion also significantly worsened patient outcomes. Patients with p53 positivity showed a median DFS of 2.5 years and an increased risk of recurrence or progression with a hazard ratio of 2.11 (95% CI: 1.59–9.81, *p*-value < 0.001). Similarly, venous invasion presented a hazard ratio of 2.84 (95% CI: 2.11–6.82, *p*-value < 0.001), substantially reducing the median DFS to 2.3 years. The most pronounced impact was observed in patients with distant metastasis, who exhibited the highest hazard ratio of 5.56, indicating a fivefold increase in risk compared to those without metastases (95% CI: 2.45–10.18, *p*-value < 0.001), and the lowest median DFS of only 1.6 years, as presented in Table 5 and Figure 2.

## 4. Discussion

### 4.1. Analysis of Findings

The findings from this retrospective cohort study highlight significant associations between lymphovascular invasion and poor disease-free survival in patients with endometrial cancer. Patients with LV invasion showed a reduced PFS with a hazard ratio of 2.18, suggesting more than double the risk of disease recurrence or progression compared to those without LV invasion. This aligns with existing literature that identifies LV invasion as a critical prognostic factor in endometrial cancer, indicating aggressive tumor behavior and a higher likelihood of metastatic spread. Similarly, the presence of high-grade tumors (grade 3–4) and genetic markers such as p53 positivity further compounded the risks, with hazard ratios significantly elevating the probability of adverse outcomes. These markers are well recognized in oncology for their role in tumor progression and resistance to conventional therapies, which was reflected in the lower median PFS reported among these patients.

The selection of surgical access methods for patients diagnosed with endometrial cancer in our study was guided by preoperative assessments of tumor characteristics suggestive of aggressive disease, such as higher clinical stage, deeper myometrial invasion, and suspected high tumor grade based on imaging and biopsy results. While the definitive presence of lymphovascular invasion can only be confirmed postoperatively through histopathological examination, the anticipation of more extensive disease influences the choice of a more radical surgical approach, such as total abdominal hysterectomy, to ensure comprehensive tumor resection.

The impact of distant metastasis was particularly stark, with the highest hazard ratio observed in the study (5.56), underscoring the severe prognostic implications of metastatic disease at diagnosis. This finding is critical, as it emphasizes the necessity for early detection and more aggressive initial management strategies in patients presenting with such high-risk features. Additionally, the study’s focus on molecular characteristics like microsatellite instability (MSI) and their association with PFS outcomes provides valuable insights into the molecular heterogeneity of endometrial cancer and its influence on patient prognosis.

Interestingly, while the number of lymph nodes removed did not differ significantly between the groups, the type of surgical intervention and the extent of residual tumor (R0 vs. R1/R2) significantly affected outcomes. Patients with LV invasion were more likely to have substantial residual tumors post-surgery, likely due to the intrinsic aggressive nature of their disease, suggesting that LV invasion status could be considered when planning the extent of surgical resection. This observation may prompt further investigation into surgical practices and could potentially lead to adjustments in surgical guidelines based on LV invasion status.

As regards long-term survival, it is also essential to consider the role of radio-chemotherapy. Though in this study we focused on the negative impacting factors for DFS at initial presentation, such as LV invasion, Arslan et al. [17] underscored the pivotal role of adjuvant radiotherapy. Arslan et al. found that adjuvant radiotherapy significantly improved 10-year DFS rates to 74.3%, identifying its absence as a key unfavorable prognostic factor. Similarly, Živković Radojević et al. [18] reported a 5-year DFS of 88.2% with the use of adjuvant brachytherapy, indicating its crucial role in management strategies for FIGO stage IA patients, particularly those at higher risk of disease progression.

In a similar manner, a study by Nora Tong et al. [19] found that the use of both chemotherapy and radiation therapy significantly improved disease-free survival and overall survival in patients with FIGO stage III endometrial cancer. Specifically, those who received both treatment modalities exhibited a 5-year DFS and OS of 58.3% and 65.2%, respectively, highlighting the importance of combined adjuvant therapies in managing advanced-stage endometrial cancer. In contrast, Alicia Smart et al. [20] focused on the efficacy of low-dose adjuvant vaginal brachytherapy in early-stage non-endometrioid endometrial cancer, reporting a 5-year recurrence-free survival of 74% and overall survival of 83%. Their findings emphasize the role of brachytherapy in reducing recurrence, particularly in patients with stage IA disease and without lymphovascular invasion, where 5-year vaginal and pelvic recurrence rates were remarkably low at 4% and 3%, respectively.

Regarding the LV invasion, which was the main focus of our study, the research performed by Reyes Oliver-Perez et al. [21] investigated the impact of lymphovascular space invasion on disease-free survival, overall survival, and patterns of recurrence in early-stage endometrioid endometrial cancer. They found that positive LVI significantly predicted distant recurrence with a hazard ratio of 2.37, emphasizing its prognostic importance. This aligns with findings from Jianzhang Wang et al.’s meta-analysis [22], which demonstrated that myometrial invasion, a correlate of LV invasion, was associated with increased risks of lymphovascular space invasion (relative risk [RR] 3.07), lymph node metastasis (LNM) (RR 4.45), and recurrence (RR 2.06). Both studies underscore the critical role of LV and myometrial invasion in predicting worse outcomes in endometrial cancer, suggesting that these factors should be integral to risk stratification and treatment planning, particularly in assessing the need for more aggressive adjuvant therapies.

Considering the varying stages of disease at diagnosis that influence DFS and OS, a study by Zeliha F Cuylan et al. [23] explored the prognostic impact of lymphovascular space invasion in stage IIIC endometrioid endometrial cancer, identifying significant predictors of decreased overall survival. They found that grade III histology, cervical stromal invasion, and myometrial invasion ≥50% were independent prognostic factors for decreased OS, with 5-year OS rates standing at 75.1%. Similarly, the Swedish Gynecologic Cancer Group study led by Karin Stålberg et al. [24] underscored LV invasion as a critical factor for lymph node metastases and survival, presenting it as having the strongest association with lymph node metastases (RR = 5.46) and significantly impacting survival outcomes (excess mortality rate (EMR) = 7.69). Both studies demonstrate the profound influence of LV invasion on the progression and prognosis of endometrioid endometrial cancer, advocating for its consideration in risk stratification and treatment planning to potentially enhance survival rates and tailored therapeutic strategies.

To reflect the latest insights from our research, it is necessary to highlight the necessity for aggressive management strategies in high-risk early endometrial cancer cases. This includes the potential benefit of concurrent chemotherapy and radiotherapy, as suggested by outcomes from the PORTEC 3 trial [25]. Such approaches could significantly improve overall survival and progression-free survival for these patients. This recommendation reinforces the importance of tailored treatment plans based on individual risk assessments to optimize patient outcomes.

Nevertheless, National Comprehensive Cancer Network (NCCN) and European Society for Medical Oncology (ESMO) guidelines emphasize the significance of lymphovascular invasion in early endometrial cancer. NCCN guidelines recommend considering adjuvant therapy for patients with LV invasion due to an increased risk of recurrence, potentially including radiation or combined modality therapy. ESMO guidelines also recognize LV invasion as a critical prognostic factor, advising that its presence should influence post-surgical management, potentially necessitating adjuvant systemic therapy based on other risk assessments.

### 4.2. Study Limitations and Future Perspectives

This study, however, is not without limitations. Its retrospective nature and single-center design may limit the generalizability of the findings. The inherent biases associated with retrospective data collection, including selection bias and information bias, could have influenced the results. Additionally, the small sample, especially of patients with LV invasion, may reduce the statistical power of the analyses conducted. Future studies with a prospective design, multicenter involvement, and larger samples are needed to validate these findings and potentially adjust clinical practice guidelines.

## 5. Conclusions

In conclusion, this study reinforces the prognostic significance of lymphovascular invasion, high-grade tumors, genetic aberrations such as p53 positivity, and the presence of distant metastasis in determining the disease-free survival of patients with endometrial cancer. The association between these factors and significantly worsened survival outcomes necessitates a tailored approach to the management of high-risk endometrial cancer patients. Implementing aggressive diagnostic and treatment strategies in this subgroup could potentially improve prognosis and survival rates. Overall, these findings contribute to the growing body of evidence that supports stratified patient management in endometrial cancer based on risk factors associated with poor outcomes.

## Figures and Tables

**Table 1:** Background characteristics of patients diagnosed with endometrial cancer with and without lymphovascular invasion.

Variables	No LV Invasion (*n* = 261)	LV Invasion (*n* = 50)	*p*-Value
Age, years (mean ± SD)	67.0 ± 11.1	68.2 ± 13.1	0.726
Age category			0.185
18–40 years	1.1%	4.0%	
40–60 years	26.1%	18.0%	
>60 years	72.8%	78.0%	
Comorbidities			0.729
Cardiovascular disease	8.1%	2.0%	
Diabetes	6.5%	4.0%	
Obesity	10.3%	6.0%	

SD—standard deviation; LV—lymphovascular.

**Table 2:** Histology and surgical management of patients diagnosed with endometrial cancer with and without lymphovascular invasion.

Variables	No LV Invasion (*n* = 261)	LV Invasion (*n* = 50)	*p*-Value
Secondary carcinoma (%)	9.58%	10.0%	0.916
Histology (%)			0.772
Endometrioid	79.3%	76.0%	
Serous	8.0%	12.0%	
Carcinosarcoma	6.9%	8.0%	
Other	5.7%	4.0%	
MSI (%)	8.4%	18.0%	0.038
p53 (%)	3.4%	14.0%	0.002
Time from diagnosis to surgery (mean ± SD)	7.79 ± 6.35	9.01 ± 7.28	0.211
Type of surgery			0.001
Total abdominal hysterectomy	25.29%	36.00%	
Laparoscopic hysterectomy	64.37%	44.00%	
Vaginal hysterectomy	1.15%	8.00%	
Robot-assisted hysterectomy	1.92%	6.00%	
Surgical access			<0.001
Abdominal access	17.24%	44.00%	
Vaginal access	69.73%	42.00%	
Laparoscopic access	3.45%	2.00%	
Combined access	0.38%	4.00%	
Residual tumor after surgery			<0.001
R0 (No residual tumor)	89.27%	62.00%	
R1 (Microscopic residual tumor)	1.15%	18.00%	
R2 (Macroscopic residual tumor)	0.77%	14.00%	

SD—standard deviation; LV—lymphovascular; MSI—microsatellite instability.

**Table 3:** Cancer staging and grading of patients diagnosed with endometrial cancer with and without lymphovascular invasion.

Variables	No LV Invasion (*n* = 261)	LV Invasion (*n* = 50)	*p*-Value
Pathological node status pN (%)			<0.001
0	48.00%	0.00%	
1	0.00%	69.73%	
2	0.00%	30.27%	
x	52.00%	0.00%	
Grade (%)			<0.001
1–2	73.46%	34.00%	
3–4	25.77%	64.00%	
Venous invasion (%)			<0.001
Yes	1.53%	64.00%	
No	98.47%	36.00%	
Distant metastasis (%)			<0.001
Yes	1.92%	22.00%	
No	95.77%	74.00%	
Number of removed lymph nodes (mean ± SD)	9.39 ± 16.55	14.43 ± 18.35	0.320

SD—standard deviation; LV—lymphovascular.

**Table 4:** Cancer outcomes among patients diagnosed with endometrial cancer with and without lymphovascular invasion.

Variables	No LV Invasion (*n* = 261)	LV Invasion (*n* = 50)	*p*-Value
Second surgery (%)	78.95% No	66.67% No	0.047
Intraoperative and immediate postoperative complications (%)	39.08% Yes	60.00% Yes	0.014
Radiotherapy (%)	49.43% No, 39.08% Yes	26.00% No, 60.00% Yes	0.003
Chemotherapy (%)	72.94% No, 22.94% Yes	28.57% No, 57.14% Yes	<0.001
Follow-up PFS (%)			0.001
Year 1	95.1%	84.6%	
Year 2	90.6%	73.3%	
Year 3	85.9%	66.0%	
Year 4	81.7%	57.4%	
Year 5	74.3%	50.8%	
Recurrence (%)	14.78% Yes	17.95% Yes	0.738
Deceased (%)	17.59% Yes	40.48% Yes	<0.001

LV—lymphovascular; PFS—progression-free survival.

**Table 5:** Factors significantly impacting DFS.

Negative Factors	PFS Median	Hazard Ratio	95% CI	*p*-Value
Lymphovascular invasion	3.2	2.18	1.56–3.04	<0.001
High-grade tumor (grade 3–4)	2.8	1.93	1.42–2.61	0.001
Microsatellite instability (MSI)	2.9	2.67	1.18–7.36	0.004
p53 positivity	2.5	2.11	1.59–9.81	<0.001
Venous invasion	2.3	2.84	2.11–6.82	<0.001
Distant metastasis	1.6	5.56	2.45–10.18	<0.001

PFS—progression-free survival; MSI—microsatellite instability.

## Data Availability

The data presented in this study are available on request from the corresponding author.

## References

[1] Bassette E., Ducie J.A. (2024). Endometrial Cancer in Reproductive-Aged Females: Etiology and Pathogenesis. Biomedicines.

[2] Staples J.N., Rauh L., Peach M.S., Baker W.D., Modesitt S.C. (2018). Endometrial cancer in an increasingly obese population: Exploring alternative options when surgery may not cut it. Gynecol. Oncol. Rep..

[3] Constantine G.D., Kessler G., Graham S., Goldstein S.R. (2019). Increased Incidence of Endometrial Cancer Following the Women’s Health Initiative: An Assessment of Risk Factors. J. Womens Health.

[4] Kuhn T.M., Dhanani S., Ahmad S. (2023). An Overview of Endometrial Cancer with Novel Therapeutic Strategies. Curr. Oncol..

[5] Restaino S., Paglietti C., Arcieri M., Biasioli A., Della Martina M., Mariuzzi L., Andreetta C., Titone F., Bogani G., Raimondo D. (2023). Management of Patients Diagnosed with Endometrial Cancer: Comparison of Guidelines. Cancers.

[6] Zheng W. (2023). Molecular Classification of Endometrial Cancer and the 2023 FIGO Staging: Exploring the Challenges and Opportunities for Pathologists. Cancers.

[7] Ogunmuyiwa J., Williams V. (2024). Emerging Advances in Endometrial Cancer: Integration of Molecular Classification into Staging for Enhanced Prognostic Accuracy and Implications for Racial Disparities. Cancers.

[8] Liu Y., Broaddus R.R., Zhang W. (2015). Identifying aggressive forms of endometrioid-type endometrial cancer: New insights into molecular subtyping. Expert Rev. Anticancer Ther..

[9] Corr B., Cosgrove C., Spinosa D., Guntupalli S. (2022). Endometrial cancer: Molecular classification and future treatments. BMJ Med..

[10] Addante F., d’Amati A., Santoro A., Angelico G., Inzani F., Arciuolo D., Travaglino A., Raffone A., D’Alessandris N., Scaglione G. (2024). Mismatch Repair Deficiency as a Predictive and Prognostic Biomarker in Endometrial Cancer: A Review on Immunohistochemistry Staining Patterns and Clinical Implications. Int. J. Mol. Sci..

[11] Anderson C., Bae-Jump V.L., Broaddus R.R., Olshan A.F., Nichols H.B. (2021). Long-term Patterns of Excess Mortality among Endometrial Cancer Survivors. Cancer Epidemiol. Biomark. Prev..

[12] Gottwald L., Pluta P., Piekarski J., Spych M., Hendzel K., Topczewska-Tylinska K., Nejc D., Bibik R., Korczyński J., Ciałkowska-Rysz A. (2010). Long-term survival of endometrioid endometrial cancer patients. Arch. Med. Sci..

[13] Shin D.W., Jung K.W., Ha J., Bae J. (2022). Conditional relative survival of patients with endometrial cancer: A Korean National Cancer Registry study. J. Gynecol. Oncol..

[14] Kasius J.C., Pijnenborg J.M.A., Lindemann K., Forsse D., van Zwol J., Kristensen G.B., Krakstad C., Werner H.M.J., Amant F. (2021). Risk Stratification of Endometrial Cancer Patients: FIGO Stage, Biomarkers and Molecular Classification. Cancers.

[15] Njoku K., Barr C.E., Crosbie E.J. (2022). Current and Emerging Prognostic Biomarkers in Endometrial Cancer. Front. Oncol..

[16] Sait K.H., Anfinan N., Sait H., Shamrani H., Sait M. (2023). Overall and progression-free survival in endometrial carcinoma: A single-center retrospective study of patients treated between 2000–2018. Ann. Saudi Med..

[17] Arslan S.A., Avcı G.G., Akkas E.A., Guney Y. (2020). Improved disease-free survival with adjuvant radiotherapy in early-stage endometrial cancer: 10-year outcome analysis. J. Contemp. Brachytherapy.

[18] Radojević M.Ž., Mujković S., Zoran P., Lončar D., Nedović N., Nedović J., Vojinović R., Dimitrijević A., Vulović T., Milosavljević N. (2023). Five-year disease-free survival in FIGO IA stage endometrial cancer patients: Tertiary institution experience in a developing country. J. Contemp. Brachytherapy.

[19] Tong N., Kumar A., Gelowitz G., Tinker A., Holloway C., Ko J. (2023). Impact of the adjuvant management and risk factors on survival in FIGO stage 3 endometrial cancer patients. Front. Oncol..

[20] Smart A., Buscariollo D., Alban G., Buzurovic I., Cheng T., Pretz J., Krechmer B., King M., Lee L. (2020). Low-dose adjuvant vaginal cylinder brachytherapy for early-stage non-endometrioid endometrial cancer: Recurrence risk and survival outcomes. Int. J. Gynecol. Cancer.

[21] Oliver-Perez M.R., Padilla-Iserte P., Arencibia-Sanchez O., Martin-Arriscado C., Muruzabal J.C., Diaz-Feijóo B., Cabrera S., Coronado P., Martín-Salamanca M.B., Pantoja-Garrido M. (2023). Lymphovascular Space Invasion in Early-Stage Endometrial Cancer (LySEC): Patterns of Recurrence and Predictors. A Multicentre Retrospective Cohort Study of the Spain Gynecologic Oncology Group. Cancers.

[22] Wang J., Xu P., Yang X., Yu Q., Xu X., Zou G., Zhang X. (2021). Association of Myometrial Invasion with Lymphovascular Space Invasion, Lymph Node Metastasis, Recurrence, and Overall Survival in Endometrial Cancer: A Meta-Analysis of 79 Studies with 68,870 Patients. Front. Oncol..

[23] Cuylan Z.F., Oz M., Ozkan N.T., Comert G.K., Sahin H., Turan T., Akbayir O., Kuscu E., Celik H., Dede M. (2018). Prognostic factors and patterns of recurrence in lymphovascular space invasion positive women with stage IIIC endometriod endometrial cancer. J. Obstet. Gynaecol. Res..

[24] Stålberg K., Bjurberg M., Borgfeldt C., Carlson J., Dahm-Kähler P., Flöter-Rådestad A., Hellman K., Hjerpe E., Holmberg E., Kjølhede P. (2019). Lymphovascular space invasion as a predictive factor for lymph node metastases and survival in endometrioid endometrial cancer—A Swedish Gynecologic Cancer Group (SweGCG) study. Acta Oncol..

[25] de Boer S.M., Powell M.E., Mileshkin L., Katsaros D., Bessette P., Haie-Meder C., Ottevanger P.B., Ledermann J.A., Khaw P., Colombo A. (2018). Adjuvant chemoradiotherapy versus radiotherapy alone for women with high-risk endometrial cancer (PORTEC-3): Final results of an international, open-label, multicentre, randomised, phase 3 trial. Lancet Oncol..

